# Understanding People’s Use of and Perspectives on Mood-Tracking Apps: Interview Study

**DOI:** 10.2196/29368

**Published:** 2021-08-11

**Authors:** Stephen M Schueller, Martha Neary, Jocelyn Lai, Daniel A Epstein

**Affiliations:** 1 Department of Psychological Science University of California, Irvine Irvine, CA United States; 2 Department of Informatics University of California, Irvine Irvine, CA United States

**Keywords:** mental health, mobile apps, mHealth, emotions, affect, self-tracking

## Abstract

**Background:**

Supporting mental health and wellness is of increasing interest due to a growing recognition of the prevalence and burden of mental health issues. Mood is a central aspect of mental health, and several technologies, especially mobile apps, have helped people track and understand it. However, despite formative work on and dissemination of mood-tracking apps, it is not well understood how mood-tracking apps used in real-world contexts might benefit people and what people hope to gain from them.

**Objective:**

To address this gap, the purpose of this study was to understand motivations for and experiences in using mood-tracking apps from people who used them in real-world contexts.

**Methods:**

We interviewed 22 participants who had used mood-tracking apps using a semistructured interview and card sorting task. The interview focused on their experiences using a mood-tracking app. We then conducted a card sorting task using screenshots of various data entry and data review features from mood-tracking apps. We used thematic analysis to identify themes around why people use mood-tracking apps, what they found useful about them, and where people felt these apps fell short.

**Results:**

Users of mood-tracking apps were primarily motivated by negative life events or shifts in their own mental health that prompted them to engage in tracking and improve their situation. In general, participants felt that using a mood-tracking app facilitated self-awareness and helped them to look back on a previous emotion or mood experience to understand what was happening. Interestingly, some users reported less inclination to document their negative mood states and preferred to document their positive moods. There was a range of preferences for personalization and simplicity of tracking. Overall, users also liked features in which their previous tracked emotions and moods were visualized in figures or calendar form to understand trends. One gap in available mood-tracking apps was the lack of app-facilitated recommendations or suggestions for how to interpret their own data or improve their mood.

**Conclusions:**

Although people find various features of mood-tracking apps helpful, the way people use mood-tracking apps, such as avoiding entering negative moods, tracking infrequently, or wanting support to understand or change their moods, demonstrate opportunities for improvement. Understanding why and how people are using current technologies can provide insights to guide future designs and implementations.

## Introduction

### Background

Mental health and wellness are common and widespread concerns. Nearly 1 in 4 people will experience a diagnosable mental health condition in their life [1], and many more will experience levels of mental distress that do not rise to clinical levels [2]. The chronic underfunding of mental health services means that not enough trained providers exist to meet the need for evidence-based mental health care. As people look for tools to help them understand and improve their mental health and wellness, they are turning to a multitude of nontraditional options, including health technologies. About two-thirds of teens and young adults [3] and nearly half of adults [4] report having used a mobile health app, with many using apps specifically for mental health and wellness. The landscape of health technology is growing rapidly; estimates suggest that over 300,000 health app exist, with over 10,000 of those for mental health and wellness [5]. One of the most common reasons people use a health app is to track some aspect of their health [6]. In the mental health and wellness space, tracking usually involves either specific tracking, which focuses on disorder-related symptoms, or general tracking, which relates to transdiagnostic factors—or those that transcend disorders or that everyone experiences to various degrees [7]. The most common transdiagnostic factor in tracking of mental health and wellness is mood. Reviews conducted in 2020 [8] and 2021 [9] have found that tracking of moods, thoughts, or behaviors is present in over half of mental health and depression apps, and is one of most common features of these apps. Thus, mood tracking appears to be a common, relevant health feature that people track through apps.

Despite the prevalence of mood-tracking features in mental health apps, most research on mood-tracking apps has focused on (1) novel system design or (2) advancing methods for mood tracking in the context of treatment for specific clinical disorders (eg, [10-12]). Less is known about people’s experience with publicly available mood-tracking apps, that is, apps that people can download directly from the app stores and start using on their own. The few studies that have explored people’s experiences with mood-tracking apps have built understanding from app store reviews [13,14], but these are limited in the type of information they contain, and also tend to come from a biased sample of users with particularly positive or negative experiences to share [5].

This study aims to fill this gap by understanding more deeply the experience and views of those who have used publicly available mood-tracking apps in their daily lives. To gain more understanding of how and why people use apps to track their moods, we conducted an interview study with people who had used or were currently using a mood-tracking app. Data were collected in a semistructured interview that enabled us to learn about people’s experiences using mood-tracking apps “in the wild,” as well as through a card-sorting task to learn about people’s reactions to screens from various publicly available mood-tracking apps. In both the interview and card-sorting task, we explored what people find useful and challenging about data entry and review approaches in existing mood-tracking apps. Our procedures, including the questions in the semistructured interviews and the types of screens selected, were guided by models of personal informatics; thus, we first discuss personal informatics including considerations of entering and tracking data. We then discuss the existing literature on mood-tracking apps to better frame our contribution to current knowledge.

### Self-Tracking and Personal Informatics

Technology designed to support people in tracking aspects of themselves and their behaviors, known as personal informatics systems [15], has become increasingly widespread. Personal informatics systems to support health and wellness are particularly common, supporting people in monitoring their physical activity [16], diet [17,18], sleep [19], and mood [14]. Research in personal informatics has primarily focused on understanding people’s needs and practices around tracking behaviors they have some direct control over, such as what and how much a person is eating or walking [20], often with the aim of making these behaviors more salient to satisfy curiosity or promote awareness [15], or to facilitate behavior change or habit formation [16].

By contrast, mood is not a behavior, rather it is a bodily and cognitive experience encompassing both physiological reactions and thoughts [21]. Accordingly, people may not have direct control over their mood and might be interested in understanding *why* certain mood states occur. This might hold some similarity to other bodily or cognitive experiences people track including chronic health conditions such as migraine [22], multiple sclerosis [23], irritable bowel syndrome [24], or recurrent bodily experiences such as menstrual cycles [25]. In these domains, people often aim to predict when an event might come (eg, when a person is due to have their next period), monitor a symptom’s frequency (eg, whether migraines are occurring more often), or learn what triggers a symptom (eg, whether a food triggers an intolerance).

Self-tracking domains vary in *what* kinds of data they collect and *how* they present that data back to the user. Aligning with the quantified self-mantra of “self-knowledge through numbers” [26], many personal informatics systems strive to collect *quantitative* and *objective* data which can be sensed and are particularly well-suited to quantification, such as steps walked or food eaten. Other apps aim to support quantification of journaled data, such as validated questionnaire scales for stress [27] and calorie lookups for foods [18]. Measurable goals can be useful for goal setting and monitoring [28], and are often visualized to promote self-reflection [15]. Other personal informatics systems instead leverage qualitative or multimedia data, such as ratings, explanations, and photos. These systems can help promote awareness such as of food choices [29]. Systems involving open-ended data can also support people in curating a record for later reflection, reminiscence, or sensemaking [30]. Researchers who contribute to the personal informatics literature often look to understand and overcome barriers to *collection* of self-tracked data and support *reflection* on such data [15]. Reflection is often supported through visual summaries or feedback to promote self-understanding or encourage desirable behaviors [31]. Systems have proposed that self-understanding and persuasion can be promoted by summarizing tracked data in natural language sentences [32], presenting data passively on the home or lock screen of a phone [33], forecasting future data based on current data [34], encoding data into abstract shapes or images [35], or encouraging reflective thought on specific events from the past [36]. However, it is unclear what data form(s) people seek and prefer for mood.

### Mood-Tracking Apps

Most research on mood-tracking apps has attempted to advance novel system design or to understand their benefits. Some examples of novel system designs include using various visual representations of mood [37], tangible modalities [11], and reducing traditional assessments of mood [38]. Although mood is tracked manually in these instances, other work is exploring whether tracking could be done automatically through passive collection of data from smartphones or wearable devices [39-41]. One way to determine the benefit of mood-tracking apps is to compare them with traditional forms of tracking, such as paper-and-pencil tracking. Comparisons between mood-tracking apps and traditional paper versions of mood assessments for individuals with mood disorders have shown similar outcomes [12,42,43]. For example, MobiMood, a mood-tracking app developed for tablet devices, was compared with traditional measures of depression, anxiety, and rumination and MobiMood was found to have good validity, while improving the ability to capture daily fluctuations in mood over those measures [44]. Other mood-tracking apps, such as Aurora and Monarca, have been reported by users to support greater awareness of their emotion and moods and provide useful information to reflect on [45,46]. However, translational barriers mean that apps designed and evaluated in research settings are rarely available on app stores. Moreover, commercially available mental health technologies vary considerably in the actual features they offer [47]. Thus, there is a need to study publicly available mood-tracking apps, and how people perceive their benefits, as people’s experiences with publicly available mood-tracking apps differ significantly from experiences with research prototypes.

Some work has begun to understand publicly available mood-tracking apps by characterizing their features. Caldeira et al [14] reviewed mood-tracking apps in light of Li et al’s model of personal informatics [15] and found that the majority of features mapped on to the collection (entry) and reflection (review) phases. For mood entry, some apps allow people to select from faces or emojis, typically labeled with text descriptors (eg, “happy,” “angry,” “content”). Others use 1 or more scales, sometimes with textual anchors (eg, “terrible,” “ok,” “great”) or without, or allow people to select from a list of words or describe their own mood in free text. For mood review, many apps use a form or list to summarize the moods a person has logged, for example, a scrolling feed of all entries or a daily list summarizing the moods a person has journaled in a day. Others provide graphs which summarize the relative frequency of different logged moods, showing how moods have varied over time; or calendars to describe the typical mood each day, week, or month; or simply surface counts of the number of times various moods have been logged. Although research has demonstrated that people like apps that facilitate mood tracking [6], little research has understood why and what features of mood-tracking apps drive this interest.

Some work has explored people’s perceptions of app features and general impressions of mood-tracking apps, and has demonstrated that tracking one’s mood might be motivated by desires to produce awareness and become more mindful of one’s own mental health [48]. Although Drake and colleagues [49] found only moderate enthusiasm for the *usefulness* of an online mood-tracking platform, participants found the ability to see one’s mood displayed over time beneficial. However, some of the features participants rated as less useful, such as automatic feedback provided about one’s mood, might have been rated lower not because that feature could not be helpful, but instead due to the feature’s implementation. Two studies, Caldeira and colleagues [14] and Widnall and colleagues [13], explored users’ experiences of a wide variety of publicly available mood-tracking apps; however, they did so by analyzing app reviews from the app stores.

In sum, past work has proposed and evaluated diverse features facilitating data entry and review for mood-tracking apps. However, this work has focused primarily on the design and feasibility of novel mood-tracking technologies, rather than on understanding why and how people are using publicly available and popular mood-tracking technologies in the real world. These novel mood-tracking technologies seldomly become available to the public after their feasibility assessments, leading to a research-to-product gap. Feedback on specific apps and app reviews are both somewhat limited in providing understanding of people’s experiences with apps. App reviews tend to offer open-ended and general reflections rather than specific aspects of app features, while reflections on single apps can relate to specific implementation challenges. Therefore, we need deeper understanding of people’s experience with various mood-tracking apps and reactions to the different ways mood-tracking features including data entry and review are implemented. We therefore sought to better understand why people who choose to use mood-tracking apps in real-world settings do so and how useful or problematic people find aspects of these apps, especially features of data entry and review. We did so by conducting semistructured interviews and a card sorting task with people who were or had previously used a mood-tracking app. We were specifically interested in answering the following research questions:

- Why and how do people use mood-tracking apps?
- What do people find useful about the approaches that mood apps take to entry and review?
- What do people find challenging about the approaches that mood apps take to entry and review?

Answering these questions will help better understand the use of mood-tracking apps in real-world contexts and might inform the design and implementation of mental health technologies to account for people’s everyday perspectives and experiences.

## Methods

### Procedure

We conducted in-person sessions with people who were currently using or had previously used a mood-tracking app. The study was approved by the Institutional Review Board at the University of California, Irvine. Consent was obtained via oral confirmation including receipt and review of a study information sheet during the in-person sessions. These sessions were performed by research team members including student trainees, masters-level research staff, and faculty with expertise in psychology and computer science/informatics. Each session consisted of a semistructured interview and a card sorting activity, split roughly evenly between the interview and the activity. Participants granted verbal consent to participate and to have the interview audio recorded. We compensated participants US $30 for their time. Combining the semistructured interview and the card sorting activity, the average total length of these sessions was 54 minutes (SD 10.1 minutes; range 24.3-68.7 minutes).

The semistructured interviews aimed to understand what participants who used mood-tracking apps hoped to gain from using them, what data they typically entered, and whether or how they used the information. We asked probing questions to further understand what motivated them to enter and review their mood data and their thoughts about their app’s approach to entry and review. Multimedia Appendix 1 provides the semistructured interview guide used for the interview portion. Prior to starting the interview study, we conducted a systematic review of publicly available mood-tracking apps to understand the types of entry and review features they contained. Although not the focus of this study, this feature review informed the development of the interview guide and screens selected for our card sorting activity. Additionally, we incorporated concepts of data entry and review from personal informatics and from previous reviews of features of mood-tracking apps [14]. We discussed these questions as a study team to arrive at an initial semistructured interview guide and iterated on the guide during training activities and practice sessions described below.

**Figure 1:**
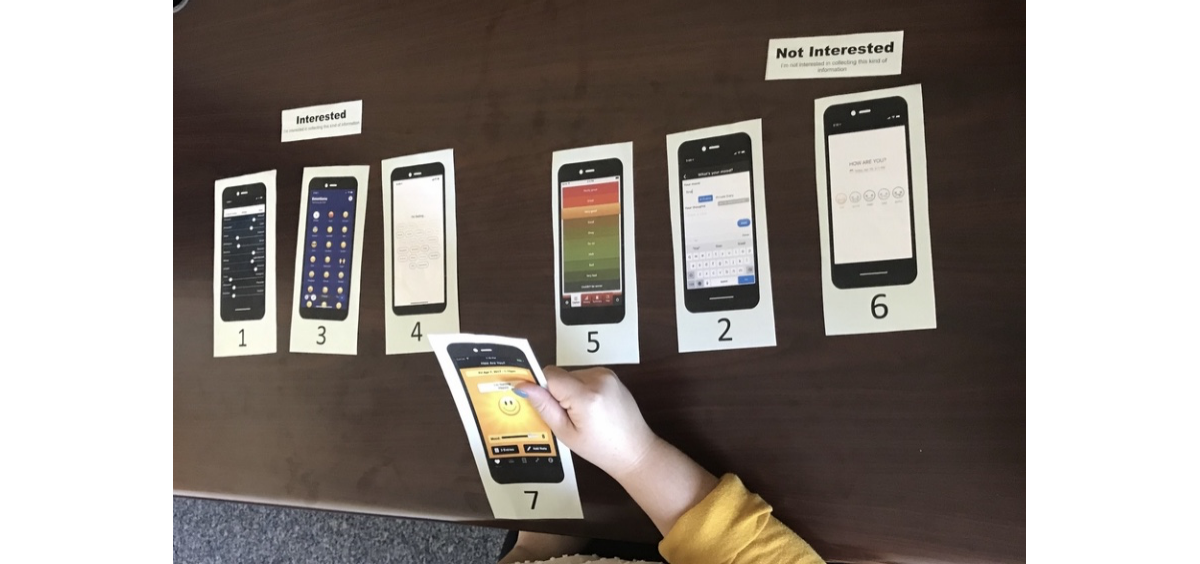
Example card sorting task demonstrating how participants sorted data entry screens into those they were “Interested” or “Not Interested” in.

The card sorting activity aimed to understand how people felt about the data entry and review methods typical of mood-tracking apps. We chose 7 data entry and 5 data review screens representative of common strategies used in commercial mood-tracking apps. We selected apps that had representative combinations of mode of entry and mood granularity (ie, number of different options for entering one’s mood) for entry and types of displays, visual representations, and features to facilitate interpretation. For example, data entry techniques included sliders with words at each endpoint, selection of relevant emojis, and open-ended text description fields. Data review techniques included feeds (ie, an updated and ongoing list of all the data entered by the user) and line charts of logged data over time, and pie and bar charts aggregating data from the past week or month. Multimedia Appendix 2 shows the data entry screens and Multimedia Appendix 3 shows the data review screens. Participants first categorized entry screens and then review screens into those they were “Interested” or “Not interested” in; participants were permitted to also create their own category (eg, “kind of interested” or “really not interested”). While sorting these cards, participants were asked to explain aloud why they found a particular entry or review screen interesting or not. We then asked participants to further categorize the screens they were interested in as “Too Specific,” “Too General,” or “Just Right.” Again, we asked participant to explain the rationale for their choices aloud. Once participants had finished sorting, we asked probing questions to further understand why participants had categorized screens in particular ways. Figure 1 displays an example of the card sorting task for entry screens.

In order to prepare members of the research team for the interviews and card-sorting task, we had several training sessions including group didactics, one-on-one interview practice, a mock session with a supervisor, supervisor participation in initial session(s), and ongoing group supervision. These sessions were facilitated by 3 members (SMS, DAE, and MN) of the lead research team having extensive and diverse experience with interviewing, including 1 PhD-level clinical psychologist (SMS), 1 PhD-level human–computer interaction researcher/ informaticist (DAE), and 1 master’s-level project manager (MN). Group didactics included 2 sessions; the first session on interviewing tips included topics such as taking effective notes, facilitating conversation, using identified probes to ask follow-up questions, and addressing difficulties that might arise in interviews, and the second session was on observing an experiencing interviewer conduct the session protocol with a mock participant. An interviewing guide was provided to all research team members following the session on interviewing tips to help support conducting the interviews, in addition to a 1-page tip sheet with examples of open and probing questions which they could refer to during interviews. All interviewers then conducted one-on-one practice sessions with another research team member that was audio recorded and later reviewed by supervisors. Portions of these practice sessions were played in group meetings to help support training on interview methods. Prior to conducting a session with a participant, each research team member conducted a mock session with a supervisor to practice completing the full session protocol and to receive feedback from a supervisor. Lastly, a supervisor attended at least one session for each team member, to observe interviewing skills with participants and to ask follow-up questions as needed. Supervisors were required to sign off on team members training in order for them to start interviewing participants without a supervisor present, and in some cases interviewers had a supervisor present for more than 1 interview. Given that all participant sessions were recorded, portions of sessions were reviewed in group supervision meetings on an ongoing basis to support consistency with the session protocol.

### Participants

We recruited 22 participants from May to August 2019 through flyers on and around our university campus, local community centers, and coffee shops, and by email to our university-affiliated research registry. Participants were required to be at least 18 years old and have used an app that allowed them to track their mood, feelings, or mental well-being for at least two weeks. Flyers and emails listed these eligibility criteria, the length of study participation, and the study compensation. Eligibility criteria were checked prior to scheduling their session, including the name of the app the participant had used. Table 1 presents participant demographics. Although our sample skewed young (mean 24.7 [SD 8.9]), participants had a range of ages; 82% (18/22) of participants were women, consistent with data suggesting that women are more likely to use health [50] and mental health apps [51]. Our sample was highly educated, with all but 1 participant indicating completion of some college and many participants having advanced degrees. Although our participants reported using a diverse set of mood-tracking apps, Daylio was commonly used with over 40% (9/22, 41%) of the participants using it. We quote participants in the “Results” section with PXX.

**Table 1:** Demographic data of participants (N=22).

Demographic		Participants, n (%)
**Gender**		
	Female	18 (82)
	Male	3 (14)
	Nonbinary	1 (5)
**Age (years**)		
	Range	18-58
	Mean (SD)	24.7 (8.9)
**Race/Ethnicity**		
	Asian	11 (50)
	White	6 (27)
	Hispanic-Latinx	2 (9)
	Mixed	3 (14)
**Education level**		
	High-school graduate	2 (9)
	Some college	10 (45)
	Bachelor’s degree	6 (27)
	Master’s degree	4 (18)
**App used**		
	Daylio	9 (41)
	Pacifica (Sanvello)	2 (9)
	Flo	2 (9)
	Moodpath	2 (9)
	Color Calendar	1 (5)
	Clue	1 (5)
	MoodPanda	1 (5)
	T2 Mood Tracker	1 (5)
	Perspective	1 (5)
	Pixels	1 (5)
	Thought Journal	1 (5)
**Length of app use**		
	>2 years	4 (18)
	>6 months	2 (9)
	>3 months	5 (23)
	>1 month	8 (36)
	>2 weeks	3 (14)
**Timing of app use**		
	Current users	16 (73)
	Past users	6 (27)

### Analytic Strategy

We analyzed 22 interviews using a bottom–up thematic analysis [52]. Interviewers composed memos following each interview, which summarized each participant’s perspective. An external company transcribed our recordings. Five members (SMS, MN, JL, DAE, and Lisa Vasquez) of the research team collectively affinity diagrammed [53] a subset of the interview transcripts to develop a codebook. Our codebook contained 13 codes (eg, desired benefit from using the app, preferred or disliked data entry methods) with 37 subcodes (eg, for self-awareness, for advice, a preference for gradients scales, a disliking for numeric scales). Two researchers (JL and Lisa Vasquez) coded the interview transcripts, discussing and refining codes with input from the rest of the research team. The sample size was determined based on reaching thematic saturation for the themes [54], which occurred after about 15 interviews. We interviewed another 7 participants to ensure no new trends emerged, and then stopped recruiting.

We also counted which categories participants sorted each entry and review screen into. Participants often created their own categories as “in between” the categories we provided or would describe screens in relation to one another (eg, “I’m more interested in card five than card four but less than the other options” [P3]). One researcher (DAE) grouped participant-made categories based on their description and discussed the results with the rest of the research team.

## Results

We examined why people seek to use mood-tracking apps and what they find beneficial and challenging about their strategies to supporting data entry and review.

### Why Do People Use Mood-Tracking Apps?

Much like other personal informatics domains, participant motivations for tracking were both intrinsic and extrinsic. Some participants began tracking due to life events, while others started due to recommendations, prompting, or suggestions from other people. Participants generally felt that mood tracking helped improve their self-awareness, promoted self-reflection, allowed them to relate their mood to other factors, and aided them in intervening or changing their mood.

Thirteen participants talked about starting to use a mood-tracking app based on life events. These events included particularly stressful times in a person’s life, as a New Year’s resolution, or due to an awareness that one’s mental health seemed to be suffering. P1 noted, “I first started using it when I was in high school when I was experiencing a lot of anxiety, and like some depression because I was really stressed out through the coursework and I thought that by tracking my mood and doing some meditation that are available in the app, it would have helped me to kind of see my emotions and see the pattern of it.”

Interestingly, no participant mentioned using a mood-tracking app in response to a positive time in their life with the goal of capturing or documenting positive emotions or feelings. Most of the events seemed to result in a desire to improve their mood or awareness of their negative moods.

Participants frequently found apps through recommendations from digital tools and social connections. Nine participants conducted their own searches of the app store, 7 participants received recommendations from other people, and 3 chose the mood app from an advertisement. These recommendations included personal and professional sources such as friends, family members, and therapists. As P21 noted, “So, someone I talked to online, she said she used it. She said it was pretty good. I wasn’t feeling very down when I started using it, I just figured it was like a cutesy little way to track how I’ve been doing over the course of the month or a year. So, I started using it.”

P20 similarly expressed using an app based on a recommendation; however, their recommendation came from a professional rather than someone online, “I’d been using actually mood apps for a while, because they were recommended by my psychiatrist. She pretty much told me was some people use journals and stuff like that, but that it’s easier to use an app just because everybody has a phone nowadays.”

Seventeen participants particularly appreciated how apps helped facilitate self-awareness and self-reflection. For example, P9 stated, “I think it’s good to reflect upon it. If it’s a lot of downs over a period of time, I want to sit and think, ‘Why was...What was the problem there?’ I think it’s a good start to just trying to be happier overall.”

When reflecting on their mood later, participant’s perspectives often changed: For example, P10 stated, “I write it as it is, exactly how I feel at that moment and then later I can look at it from a different perspective when I’m not angry anymore and see like was that worth it? I can see a different perspective on my emotions from a different time.”

Although apps tended not to provide support for identifying correlations, participants were still able to use their apps to identify relationships between their mood and contextual factors. Participants noted finding potential triggers for their mood including people, events, and aspects of one’s physical health. P12 reflected that the mood-tracking app she used helped: “basically, tracking my actions, seeing what I’m surrounding my environment with, and if I can eliminate any negative...just basically anything that’s not good for me, I see. It’s basically math. You see what you’re doing to have a better, healthy lifestyle. You have to subtract what’s not adding to that.”

Eight participants talked about insights they uncovered through these relationships and saw correlations which suggested specific triggers for their moods. For example, P11 stated, “I realized through tracking my emotions and through looking at the correlation and everything of when it was happening. I realized that, first of all, I was getting stressed and unhappy and just not doing well too much overall, and especially during the PMS phase.”

Although participants often aimed to better understand what led to negative mood states with the hope of potentially being able to alleviate or avoid those negative moods, 6 participants reported that they did not like to record or see indications of negative moods. Although participants appreciated relating negative moods to other information, the dislike of recording or seeing one’s negative mood presented challenges to realizing these benefits. As P3 stated, “Yeah, the thing is whenever I had a bad experience, I didn’t want to put it in the app. I just didn’t feel an inclination to open the app and put it in there, I just wanted to lay there and cry. You don’t use it for the bad times. Put all the good times on it.”

Participants also appreciated how mood-tracking apps facilitated conversations in their daily life, including with friends, family members, and professionals. Eight participants noted the use of mood-tracking apps with formal mental health support. P1 discussed her use of a mood-tracking app with her therapist: “I was seeing a therapist at the time...And she was the one who actually told me to look at the monthly progress with the app, so I can get a better picture of how I felt because I didn’t have bad weeks all the time. It’s just one here and there.”

Conversations with other people about mood-tracking apps helped provide ideas on how and why to use the app and added meaning or perspective when interpreting the data. P11 talked about the ability to use this information to have more meaningful conversations with her mom: “I feel she’s someone who has a hard time understanding other people’s emotions and mental health kind of stuff unless she has direct evidence of things. Which is unfortunate, but also I found it beneficial to be able to say, ‘Hey, look at this. This is happening during these times. There is evidence here now.’ I think that [the app] was helpful to kind of help her to understand as well.”

Social features within apps also helped a few participants normalize their experience. For example, P9 used MoodPanda, and appreciated that she could see the mood descriptions that others wrote in the app: “I mean, it’s just also not a feeling of aloneness, just seeing other people’s problems. I mean, I kind of get that, that kind of thing. I know that if I feel a certain way, it’s just a reminder of, oh, there’s other people. I’m not the first one to feel this way.”

### What Do People Like About Entry and Review in Mood-Tracking Apps?

Although we focused our questions and analysis to identify the functional benefits of entry and review, participants did comment on appreciating aspects such as aesthetics. Participants also frequently mentioned their preferences depending on other features or properties of mood-tracking apps, such as the inclusion of social features or the ability to preserve privacy.

With respect to data entry, participants seemed to appreciate simplicity in conjunction with flexibility. Participants disliked when they felt overwhelmed by the number of options of moods and emotions to track, but also wanted to be able to capture the nuance in the moods. Eight participants noted that sliders were a particularly effective way to balance nuance and options; for example, P13 stated, “same thing I said before. I like the fact that there’s a dial, a spectrum that lets you pick somewhere along the range.”

P10 similarly reflected on the benefits of sliders, noting that they are able to capture that moods vary in intensity in addition to valence, and that the discrete emotions could be mixed or felt to varying degrees: “it’s not just clicking Tired or Energetic, but it’s like on a gradient. I think that’s interesting...Because you don’t always feel completely stressed or completely relaxed. Sometimes you feel like a little bit of both or in between, so I think the gradient is important to differentiate that feeling.”

Participants thought customization could balance the desire for simplicity and flexibility. For example, P7 stated: “I prefer the having the sort of structure of having a few moods to choose from, and then if the user doesn’t really relate to those moods, then they can add their own because that adds a layer of customization now also, like sort of a personal connection, they specifically feel this way so they can put them down.”

**Figure 2:**
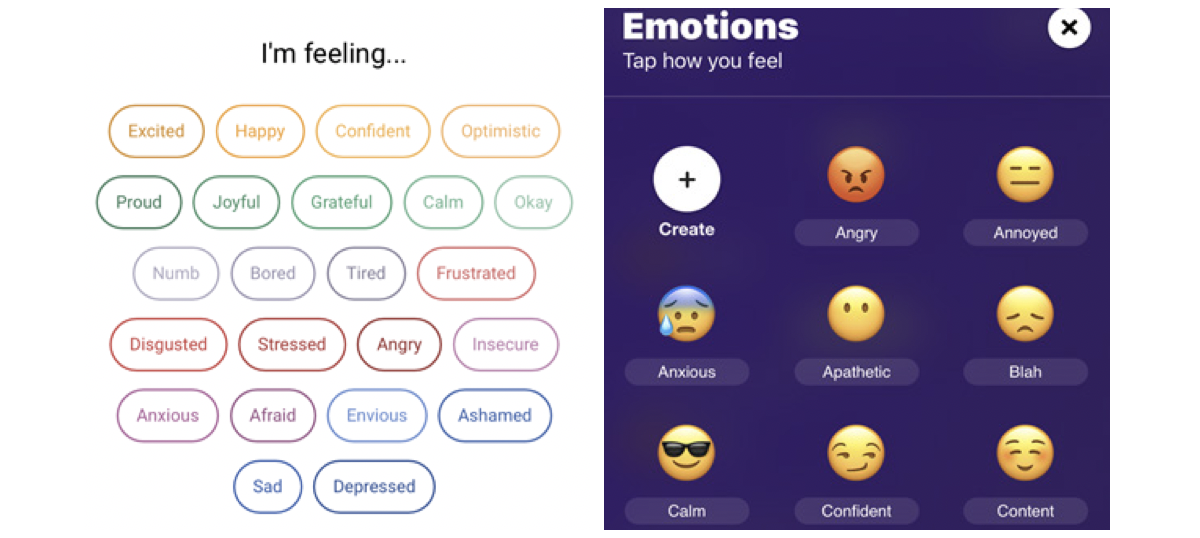
Participants preferred entry screens which allowed them to select words (Youper, left) or emojis that represent words (Mood - Journal & Anxiety Chat, right).

These preferences toward simple, but flexible entry methods were also reflected in the card sorting task. Four of the 7 data entry techniques were categorized by most of the participants (59% [13/22]-73% [16/22]) as methods they would be interested in using to enter data. All of the well-regarded screens supported multiple ways of identifying or describing moods, such as by using sliders for multiple mood states, having multiple emojis or words that could be used to represent moods, or combining emojis and words in a simple emotion scale. The 2 data entry strategies that participants found “just right” presented multiple words or emojis and allowed participants to endorse which they were experiencing (Figure 2). P12 appreciated the simplicity of entry in these screens, saying: “It’s very brief like Color Calendar. I just pick an emoji, and that’s it? Like I said, that sake of condensation. That’s very appealing.” Such screens often reduced the cognitive complexity of tracking as P16 noted: “But, I feel like it’s just right in that it has four choices so you don’t have to think about it too much. The language is accessible, again, to a casual user and if you do have diagnosis, it has a sense of humor about it.”

By contrast, screens that were less well-regarded often felt inflexible or inappropriate. For example, P15 noted, “I’m not a robot, so I just can’t go off of this scale.”

**Figure 3:**
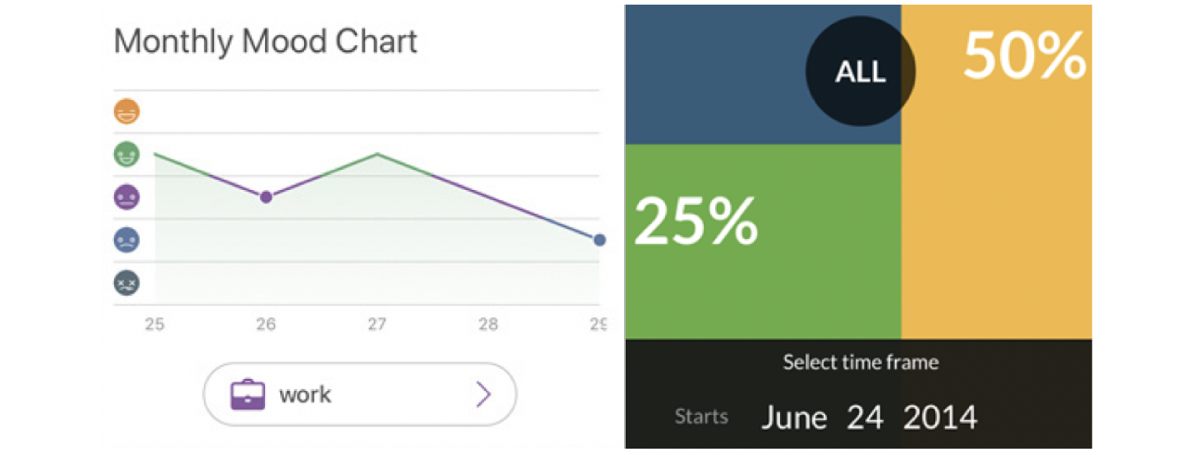
Participants preferred review screens which allowed them to see trends in their mood over time (Daylio, left) to ones which aggregated time ranges (Mood Meter, right).

Whereas P19 indicated a specific dislike for numbers: “Oh rating by numbers, I don’t think I would use that.” This further reinforces the preferences we saw for flexibility and multiple ways to enter data. In the card sorting task, the 2 data review screens that participants were most interested in used a line graph (14/22, 64% of participants) and a feed (12/22, 54%) to summarize the mood entries a person had journaled. These approaches helped capture not only what moods occurred, but when, and also each mood in relation to other entries (Figure 3). For example, of a screen with a line graph, P20 said, “I like it. It shows you in a graph how your mood goes up and down…it shows you the days in a row how you’ve been feeling like that, so you can look back and see what you can do or what you were doing that made you feel that certain type of way for those couple of days.”

P12 appreciated how a screen with a feed could help her recall what triggered the mood on a specific day: “This is more specific on the day, like off the bat I can see, and then recall, ‘Oh maybe this day, I’m doing something.’ This I would go into and see, ‘Oh what did I write down that day?’”

### What Do People Find Challenging About Mood Entry and Review?

Many participants had concerns about how meaningful or representative the mood data they logged felt to them. Some of these concerns related to how the app presented information back to them, although other participants had deeper concerns around whether the way they entered mood data might diminish its value.

Although 1 participant (P11) indicated that she was comfortable drawing inferences from the tracked data, it was much more common for participants to express discomfort or confusion. Nine participants commented on different features of the app that made interpretations from the data challenging, including an overreliance on displays that required effort to scroll or search through, or displays that merely presented data without summary or interpretation. P2’s statement was representative of other participant perspectives: “I don’t really know how to analyze it. Maybe if they gave me a method they’re like, ‘Oh, count the days you did this, divide by that.’ I could have done it - numbers, or a chart or something more mathematical, because I don’t really know how to approach this, I don’t how to do it.”

Overall, participants noted that although the apps provided the opportunity for them to collect a lot of information about their emotional life, there was little guidance to help them translate that information to something valuable. As P16 said: “I have confirmation that I am feeling shitty. But, it doesn’t really help you do anything about it.”

Not surprisingly, as 13 participants had started tracking in response to negative events or in attempts to improve their mood, participants wanted to see more interventional components in mood-tracking apps. For example, P7 stated: “I think it would be cool if it sort of had a bit more than just your own input like it took your input. Not only does it compile it into data but sort of give you solutions or recommendations based on the data that you provide.”

From this vantage point, it seemed that people wanted to see how mood-tracking apps could better help them manage and improve their own mood. Relatedly, participants also voiced concern as to whether the data collected related to mood could actually be useful, given that mood and emotions are complex phenomena that are difficult to capture and quantify. As P9 summarized: “I don’t think any app can realistically capture the human feelings.”

Six participants noted that tracking one’s mood just once a day may not effectively represent the full depth of their emotional experience or may miss important moments that would be useful to track and understand. As one example, P2 said: “one of the problems I had with it was my mood changes a lot throughout the day. I wake up and I’m in a good mood, and then 20 minutes later I’m in a bad mood, and then I’m in a good mood, and it doesn’t account for that. So, it didn’t really...I don’t know. It was kind of not helpful in that aspect.”

Many mood-tracking apps only allow the users to enter their mood once per day. However, even those that allow tracking multiple times per day may not strongly encourage that because multiple reminders and repeated entries over a day increase user burden. They might then fail to capture actual variance in some people’s mood. As P17 stated, “Mine doesn’t move too often. There’s a better way to present that information. A line graph doesn’t...Maybe for someone who’s really cycling, sure. But for me when I’m more like this, it doesn’t make much sense.”

Participants commonly reported that they would track their mood at a regular time every day, as P5 stated, “At the end of the day usually....Usually before I sleep too, because it gives me the whole day to just think about how my day went and just reflect on my day.” P7, however, noted a challenge with this in terms of what could be learned “what I’ve learned recently is that usually at 8:00 pm I’m feeling really good, but sometimes later at night, I can feel a little worse about myself, my mood can go down a little. And that doesn’t...the mood having only one daily mood can sort of ignore or doesn’t focus on the fact that my mood changed later on.”

Many apps summarize moods logged over days or weeks. Given that participants tended to only enter their mood a few times per day or less, they wondered whether the summaries presented from their data are actually meaningful. P10 stated: “You have statistics. You can tell basically how often you’re feeling happy on days, but it wasn’t right.”

In the card sorting activity, half or more of participants (50% [11/22]-59% [13/22]) indicated that they would not be interested in using the 3 data review screens which used calendars, stacking bar graphs, and pie charts to summarize the days or the amount of time they expressed a particular mood (Figure 3) because these screens tended to aggregate how frequently a mood level or mood term was indicated at varying internals (eg, day, week, or month). For example, P3 disliked how a screen summarized the percentage of entries which fit a mood category (“green” for good moods, “red” for bad): “It just shows these are all of the moods you felt during all this time…I guess not showing a trend, or specific days. You don’t know what days you felt yellow, or what days you felt green.” P19 felt similarly about a stacked bar chart: “again, it’s labeling my whole month as something. I’m not sure how I feel about that. I don’t think there’s been a month in my life where I’ve followed one trend.”

## Discussion

### Principal Results

Our findings provide an overview of what people gain from the use of mood-tracking apps that they adopted as well as the shortfalls or challenges in such tools. In general, our participants turned to mood-tracking apps to increase self-awareness or self-reflection, especially during challenging times in their lives. Participants liked simple screens for data entry and appreciated opportunities for review that linked their information, such as moods and triggers, to produce insights, but desired opportunities for customization around how such information was linked. Furthermore, our participants disliked the high burden of mood tracking and the lack of opportunities to gain self-awareness when data were sparse. Many of our participants therefore questioned the usefulness or accuracy of insights. Table 2 highlights key findings from our thematic analysis.

We highlight some potentials for mood tracking through technology, leading to design challenges and implications for future digital mood-tracking tools including (1) understanding the dynamics of mood; (2) customizability and personalization; and (3) undersupported tracking methods. As mood is an important component of mental health and wellness, these implications also have relevance to creating digital tools that can help people understand and manage their mental health and wellness.

**Table 2:** Key findings from our thematic analysis.

Research question	Key findings
Why do people use these mood-tracking apps?	Life events which triggered stressful or challenging moments motivated some participants, while others sought out mood tracking for greater self-awareness and self-reflection.
What do people find useful about the approaches that mood apps take to entry and review?	Most participants preferred data entry screens with fewer options, but which supported the ability to customize what they logged. For data review, participants wanted to be able to compare a logged mood with previous entries, preferring graphs and feeds which enabled this.
What do people find challenging about the approaches that mood apps take to entry and review?	The burden of mood tracking led participants to track once per day (or less often), and they questioned how well that mood reflected the moments they did not track and whether visualizations of that data were representative. They also found it challenging to identify if or how their mood improved based on the trends they observed.

### Understanding the Dynamics of Mood

People’s moods fluctuate and asking people how they feel at a moment may not hold much value as an evaluation [55], especially if only done once a day. Rather than journaling and reflecting on their mood in a particular moment, participants were more interested in patterns, connections, or insights. However, generating patterns, connections, or insights about one’s mood requires sufficient data collected across time that can capture the dynamics of how a person’s mood persists or changes over time, and can reveal actual, rather than illusory, correlations. Furthermore, understanding the dynamics of a person’s mood could help them understand whether moments reflect highs or lows. Mood tracking, therefore, could help unpack the extreme values that a person experiences and better support ways for people to track and understand this information.

Psychological research has demonstrated that mood variability, or the variance in one’s mood over regular intervals, is a stable trait that varies across individuals and as such might be able to be captured and represented when one starts tracking [56]. In the practice of mental health treatments, mood tracking usually begins with a short period of intense tracking to better identify one’s variability and patterns [57]. Similar approaches of intense tracking over short periods have been used to help people identify food intolerances [24] and make sleep recommendations [19]. Mood-tracking apps, however, typically do not vary their entry or review processes by incentivizing more intense tracking when beginning a program. Many health trackers do enjoy a novelty period, in which people engage in more frequent and regular use immediately after starting to use a health tracking technology [58]; however, this novelty period is based on people’s motivations rather than technological features. Intentionally structuring intense and maintenance periods of mood self-tracking could better support developing an overall understanding of a person’s mood tendencies.

In addition to variance, an important aspect of mood dynamics is people’s highs and lows. Participants in our study reported a hesitation to record extreme values when using scales or intense negative moods when using words or emojis, in part to avoid viewing these moods when reviewing their data. Similar challenges have been reported in other tracking domains, such as food, where tracking instances of making unhealthy choices might produce guilt or shame [17]. In experiential tracking domains such as fertility, people often stop tracking when the data only present struggles or lack of improvement [59]. It might be possible for data entry features to normalize or contextualize extremes, especially intense negative mood states. For example, mood-tracking apps could trigger a follow-up notification to better understand the duration of such moods, in the same way a friend or family member might check-in on a person who disclosed to them feeling particularly bad. Gathering deeper nuance during negative mood states to unpack valence as well as intensity (ie, how bad and how strong is the emotion), or providing normative data about how common such moods are either compared with one’s own or others’ data, may help encourage users to track these intense mood states. Other experiential domains may similarly benefit from normalizing lack of success or improvement.

### Customizability and Personalization

Mood is ultimately a subjective phenomenon, with potential variations in what it means across people. This is different from other health-related aspects that might be tracked objectively such as weight, calories, or steps. Although a step may vary slightly from person to person (eg, stride length, time to take a step), there is general agreement as to whether a person took a step or not. People strive for relatively accurate accounts of their experience through tracking activities [16,60], but accuracy in mood is challenging to achieve given its subjective nature. Furthermore, people vary in the language they use to describe their moods; customizability could address these issues.

Our findings parallel Ayobi and colleagues’ [61] work noting considerable variability in the way people tracked their habits and mood using paper bullet journals. Similarly, our participants had varied preferences for granularity in mood-tracking apps and the way to enter information such as emojis, words, numbers, or scales. Although participants often shared general interest in screens in our card sorting task, they varied in whether they characterized data entry screens as “too general,” “too specific,” or “just right” or data review screens as “too simple,” “too complicated,” or “just right” for review. Many apps include ways to customize and our participants reflect positively on customizability as a way to balance simplicity and flexibility. However, customizing features also requires more effort on the part of the user. This amount of effort is worrisome given that many people tend to not use features that allow customization when it fails to fit into their workflow or it requires extra time to explore the app [62].

Although customizability will help a variety of people meet their needs with an app, it is worth noting that customization is often limited to specific aspects of entry or review. For example, many mood-tracking apps allow users to customize the specific word or image used for a mood or how many different moods are displayed. Less customizability exists in terms of the form of entry. In the same way that some people find value in food journaling through calorie-driven apps, while others instead prefer using food photos to remain mindful of food choices, such variability might be useful for moods. It is worth considering whether novel apps could be developed to support diverse approaches to data entry and review. Under supported approaches to entry and review are prime opportunities for exploring and contributing new designs.

### Undersupported Tracking Methods

Given that few participants tracked their mood more than once a day, if even that, it is worth understanding how mood-tracking apps could be designed in ways to facilitate self-reflection and insight from such sparse data. Mood-tracking apps often attempt to interpret data on the assumption that they represent a complete record of a person’s mood. It is worth considering how reflection could be promoted in a mood-tracking app which explicitly promotes once-a-day mood tracking, or an app which aims to support logging during just the “difficult” moments that people appear interested in understanding yet are hesitant to track. This might be accomplished by having people reflect on their day overall rather than their mood in the moment, or have people rate their highest and lowest moods that occurred on a given day or since the last assessment, perhaps even identifying when they occurred.

Alternatively, further research could leverage how to encourage people to journal their moods more often, or to add more details when they do log. Mood-tracking apps could leverage classic techniques examined in other personal informatics domains, such as journaling from the phone lock screen [63,64], leveraging notifications [65], experience sampling and day reconstruction methods [66], or passive sensing [39,40]. When people do decide to track their current mood, conversational agents or on-screen prompts could also encourage people to reflect and log on their moods for earlier in the day, week, or month to facilitate creation of more representative mood histories. However, care needs to be taken to ensure such strategies do not interfere with people’s general desire for simple and flexible approaches to mood tracking.

Past work has demonstrated that people find photographs to be an especially useful way to track one’s mood [67]. However, commercial apps tend not to leverage this, instead using scales, emojis, or text for mood entry. People frequently take pictures of a variety of mood-relevant events using their phones, such as photos of joyful moments with family members or of stressful whiteboard meetings at work. Leveraging these photos in mood-tracking apps might be another way to maximize information while reducing burden. Sharing pictures and other visual content is an important part of various technologies, especially social technologies such as social media [68]. As it has been noted that one affordance of technology for mental health purposes is the ability to be deeply integrated into people’s daily lives [69], mood-tracking technologies might leverage this affordance to design better tracking experiences.

### Limitations

Our sample was predominantly women, however, given that we recruited people who had previously used mood-tracking apps and that women tend to use health and mental health apps [50,51], our demographics track with the general rates of adoption of such apps. We also oversampled for college students, with 45% (10/22) of our sample currently in college. College students are more likely to be tech-savvy, early adopters of technology, and may have different experiences or motivations to use these apps than the general population. However, a majority of our sample consisted of noncollege students, as we also recruited from the broader community. All participants were local due to sessions being conducted in-person. We also note that overall, our participants had relatively high levels of education and therefore might have higher levels of data literacy. Therefore, their preferences—including what they found helpful or problematic—might differ from people with less education or lower data literacy. Indeed, past research has demonstrated that people from some backgrounds find current mood-tracking tools less usable, in the case of this study those from low-income and traditionally minoritized racial and ethnic groups [70]. Further challenges around stigma, literacy, and access may prevent other demographic groups and backgrounds from getting the desired benefit from mood tracking.

Our participants’ experience with mood-tracking apps may not be representative of wider experiences. For example, many of our participants had used Daylio, which may not hold in other populations. We attempted to mitigate the limitation of the specific app a participant had used by showing participants multiple apps within our card sorting task and getting their feedback. A few screens did come from Daylio, which a few participants indicated preference toward simply due to familiarity. More often, participants weighed whether Daylio, or whichever app they had used, would be more or less helpful than the app they were considering in the activity. Most of our participants (16/22, 73%) were currently using mood-tracking apps, whereas others were past users; as such, the information shared might vary based on their ability to recall mood-tracking apps.

The benefits our participants reported were based on their perceived benefits and we did not test whether or not changes were actually accrued in these constructs over time. Nevertheless, future work could formally evaluate whether mood tracking leads to better understanding of one’s mood or the causes and consequences of one’s mood. Our results and findings should be considered in light of these limitations.

### Future Directions

As the number of technologies for mental health and wellness continue to expand, we are likely to see an increased interest in technologies that address broad aspects of mental health such as mood. We examined the perceptions that people held as to how such mood-tracking apps benefited them, but future work could test such benefits experimentally by either assigning people to use mood-tracking apps or manipulating the conditions under which they used them (eg, looking at entering and reviewing data or the number of times per day a person might be prompted to record their moods). Other work could explore the design of mood-tracking technologies from the perspective of how people currently use technologies generally and mood-tracking apps specifically. For example, mood-tracking capabilities might be integrated into widely used platforms such as messaging or social media to facilitate support and connectedness around mood. Another option would be to build mood-tracking apps optimized for once a day tracking as discussed.

Our findings also highlighted that people’s interest in mood-tracking apps was often driven by life events or circumstances and as such we should better understand how the social and cultural factors in people’s everyday lives impact perspectives and desired features of mood-tracking apps, specifically and mental health technologies. Technology use for the purpose of understanding and promoting one’s mental health needs to be placed into the context of people’s lives and the broader way they use technology. Some work has explored the way individuals with depression use technology and social media for emergent practices of self-regulation [71], and we could better understand emergent practices of self-insight and self-knowledge with regard to various affective processes including mood.

### Conclusion

In this paper, we explored mood-tracking apps from the vantage point of people’s experiences with apps they adopted in real-world settings. We found people often turned to these apps during shifts in their lives—such as negative events or changes in their mental health—with the desire to gain insight and potentially support self-awareness and behavior change. We found various instances in which these apps provided useful support for these goals, such as visualizations (figures or calendars) to help illustrate trends. However, we also found other instances in which these apps fell short, including a lack of recommendations or suggestions to support data interpretation and limited intervention features. Therefore, although mood-tracking apps have helped translate concepts from mood and emotion theories into widely available digital tools, various aspects of their design could be improved to help create tools that people would find more useful.

## Appendix

Multimedia Appendix 1 Semi-structured interview guide.

Multimedia Appendix 2 Data entry screens for card sorting task.

Multimedia Appendix 3 Data review screens for card sorting task.

## References

[1] Substance Abuse and Mental Health Services Administration (2019). Key substance use and mental health indicators in the United States: Results from the 2018 National Survey on Drug Use and Health (HHS Publication No. PEP19-5068, NSDUH Series H-54).

[2] Horwath E, Johnson J, Klerman G, Weissman M (1994). What are the public health implications of subclinical depressive symptoms?. Psych Quart.

[3] Rideout V, Fox S, Peebles A, Robb MB (2021). Coping with COVID-19: How young people use digital media to manage their mental health.

[4] Krebs P, Duncan DT (2015). Health App Use Among US Mobile Phone Owners: A National Survey. JMIR Mhealth Uhealth.

[5] Neary M, Schueller SM (2018). State of the Field of Mental Health Apps. Cogn Behav Pract.

[6] Rubanovich CK, Mohr DC, Schueller SM (2017). Health App Use Among Individuals With Symptoms of Depression and Anxiety: A Survey Study With Thematic Coding. JMIR Ment Health.

[7] Kring AM, Sloan DM (2009). Emotion regulation and psychopathology: A transdiagnostic approach to etiology and treatment.

[8] Qu C, Sas C, Daudén Roquet Claudia, Doherty G (2020). Functionality of Top-Rated Mobile Apps for Depression: Systematic Search and Evaluation. JMIR Ment Health.

[9] Lagan S, D'Mello R, Vaidyam A, Bilden R, Torous J (2021). Assessing mental health apps marketplaces with objective metrics from 29,190 data points from 278 apps. Acta Psychiatr Scand.

[10] Khue L, Ouh E, Jarzabek S (2015). Mood self-assessment on smartphones.

[11] Lee K, Hong H (2017). Designing for self-tracking of emotion and experience with tangible modality.

[12] Nahum M, Van Vleet TM, Sohal VS, Mirzabekov JJ, Rao VR, Wallace DL, Lee MB, Dawes H, Stark-Inbar A, Jordan JT, Biagianti B, Merzenich M, Chang EF (2017). Immediate Mood Scaler: Tracking Symptoms of Depression and Anxiety Using a Novel Mobile Mood Scale. JMIR Mhealth Uhealth.

[13] Widnall E, Grant CE, Wang T, Cross L, Velupillai S, Roberts A, Stewart R, Simonoff E, Downs J (2020). User Perspectives of Mood-Monitoring Apps Available to Young People: Qualitative Content Analysis. JMIR Mhealth Uhealth.

[14] Caldeira C, Chen Y, Chan L, Pham V, Zheng K (2017). Mobile apps for mood tracking: an analysis of features and user reviews.

[15] Li I, Dey A, Forlizzi J (2010). A stage-based model of personal informatics systems.

[16] Consolvo S, Everitt K, Smith I, Landay J (2006). Design requirements for technologies that encourage physical activity.

[17] Cordeiro F, Epstein D, Thomaz E, Bales E, Jagannathan A, Abowd G (2015). Barriers and negative nudgese xploring challenges in food journaling.

[18] Tsai CC, Lee G, Raab F, Norman GJ, Sohn T, Griswold WG, Patrick K (2007). Usability and Feasibility of PmEB: A Mobile Phone Application for Monitoring Real Time Caloric Balance. Mobile Netw Appl.

[19] Daskalova N, Metaxa-Kakavouli D, Tran A, Nugent N, Boergers J, McGeary J (2016). SleepCoacher: A personalized automated self-experimentation system for sleep recommendations. https://dl.acm.org/doi/10.1145/2984511.2984534.

[20] Choe E, Lee N, Lee B, Pratt W, Kientz J (2014). Understanding quantified-selfers' practices in collecting and exploring personal data.

[21] Russell J (2003). Core affect and the psychological construction of emotion. Psychol Rev.

[22] Schroeder J, Chung C, Epstein D, Karkar R, Parsons A, Murinova N, Fogarty J, Munson S (2018). Examining self-tracking by people with migraine: goals, needs, and opportunities in a chronic health condition. Proceedings of the 2018 Interactive Systems Conference.

[23] Ayobi A, Marshall P, Cox A, Chen Y (2017). Quantifying the body and caring for the mind: Self-tracking in multiple sclerosis. Proceedings of the 2017 CHI Conference on Human Factors in Computing Systems.

[24] Karkar R, Schroeder J, Epstein D, Pina L, Scofield J, Fogarty J, Kientz J, Munson S, Vilardaga R, Zia J (2017). Tummytrials: a feasibility study of using self-experimentation to detect individualized food triggers. Proceedings of the 2017 CHI Conference on Human Factors in Computing Systems.

[25] Epstein D, Lee N, Kang J, Agapie E, Schroeder J, Pina L, Fogarty J, Kientz J, Munson S (2017). Examining menstrual tracking to inform the design of personal informatics tools. Proceedings of the 2017 CHI Conference on Human Factors in Computing Systems.

[26] Wolf G (2009). Know thyself: Tracking every facet of life, from sleep to mood to pain, 24/7/365. Wired Magazine.

[27] Wang R, Chen F, Chen Z, Li T, Harari G, Tignor S, Zhou X, Ben-Zeev D, Campbell A (2014). StudentLife: assessing mental health, academic performance and behavioral trends of college students using smartphones. Proceedings of the 2014 ACM International Joint Conference on Pervasive and Ubiquitous Computing.

[28] Locke EA, Latham GP (2002). Building a practically useful theory of goal setting and task motivation. A 35-year odyssey. Am Psychol.

[29] Epstein D, Cordeiro F, Fogarty J, Hsieh G, Munson S (2016). Crumbs: Lightweight daily food challenges to promote engagement and mindfulness. Proceedings of the 2016 CHI Conference on Human Factors in Computing Systems.

[30] Elsden C, Durrant AC, Kirk DS (2016). It's just my history isn't it?: Understanding smart journaling practices. Proceedings of the 2016 CHI Conference on Human Factors in Computing Systems.

[31] Baumer E, Khovanskaya V, Matthews M, Reynolds L, Sosik V, Gay G (2014). Reviewing reflection: on the use of reflection in interactive system design. Proceedings of the 2014 Conference on Designing Interactive Systems.

[32] Epstein D, Cordeiro F, Bales E, Fogarty J, Munson S (2014). Taming data complexity in lifelogs: Exploring visual cuts of personal informatics data. Proceedings of the 2014 Conference on Designing Interactive Systems.

[33] Lane N, Mohammod M, Lin M, Yang X, Lu H, Ali S, Doryab A, Berke E, Choudhury T, Campbell A (2011). Bewell: A smartphone application to monitor, model and promote wellbeing.

[34] Hollis V, Konrad A, Springer A, Antoun M, Antoun C, Martin R, Whittaker S (2017). What Does All This Data Mean for My Future Mood? Actionable Analytics and Targeted Reflection for Emotional Well-Being. Human–Computer Interaction.

[35] Snyder J, Murnane E, Lustig C, Voida S (2019). Visually encoding the lived experience of bipolar disorder. Proceedings of the 2019 CHI Conference on Human Factors in Computing Systems.

[36] Isaacs E, Konrad A, Walendowski A, Lennig T, Hollis V, Whittaker S (2013). Echoes from the past: how technology mediated reflection improves well-being. Proceedings of the SIGCHI Conference on Human Factors in Computing Systems.

[37] Schreiber M, Jenny GJ (2020). Development and validation of the ‘Lebender emoticon PANAVA’ scale (LE-PANAVA) for digitally measuring positive and negative activation, and valence via emoticons. Personality and Individual Differences.

[38] Torkamaan H, Ziegler J (2020). Mobile mood tracking: An investigation of concise and adaptive measurement instruments. Proc. ACM Interact. Mob. Wearable Ubiquitous Technol.

[39] Sükei Emese, Norbury A, Perez-Rodriguez MM, Olmos PM, Artés Antonio (2021). Predicting Emotional States Using Behavioral Markers Derived From Passively Sensed Data: Data-Driven Machine Learning Approach. JMIR Mhealth Uhealth.

[40] Yan S, Hosseinmardi H, Kao H, Narayanan S, Lerman K, Ferrara E (2020). Affect Estimation with Wearable Sensors. J Healthc Inform Res.

[41] Zulueta J, Piscitello A, Rasic M, Easter R, Babu P, Langenecker SA, McInnis M, Ajilore O, Nelson PC, Ryan K, Leow A (2018). Predicting mood disturbance severity with mobile phone keystroke metadata: A BiAffect digital phenotyping study. J Med Internet Res.

[42] Kumar A, Wang M, Riehm A, Yu E, Smith T, Kaplin A (2020). An Automated Mobile Mood Tracking Technology (Mood 24/7): Validation Study. JMIR Ment Health.

[43] Torous J, Staples P, Shanahan M, Lin C, Peck P, Keshavan M, Onnela J (2015). Utilizing a personal smartphone custom app to assess the Patient Health Questionnaire-9 (PHQ-9) depressive symptoms in patients with major depressive disorder. JMIR Ment Health.

[44] Church K, Hoggan E, Oliver N (2010). A study of mobile mood awareness and communication through mobimood. Proceedings of the 6th Nordic Conference on Human-Computer Interaction: Extending Boundaries.

[45] Gay G, Pollak JP, Adams P, Leonard JP (2011). Pilot study of Aurora, a social, mobile-phone-based emotion sharing and recording system. J Diabetes Sci Technol.

[46] Bardram J, Frost M, Szántó K, Faurholt-Jepsen M, Vinberg M, Kessing L (2013). Designing mobile health technology for bipolar disorder: a field trial of the monarca system. Proceedings of the SIGCHI Conference on Human Factors in Computing Systems.

[47] Huguet A, Rao S, McGrath PJ, Wozney L, Wheaton M, Conrod J, Rozario S (2016). A systematic review of cognitive behavioral therapy and behavioral activation apps for depression. PLoS One.

[48] Kelley C, Lee B, Wilcox L (2017). Self-tracking for mental wellness: Understanding expert perspectives and student experiences. Proceedings of the 2017 CHI Conference on Human Factors in Computing Systems.

[49] Drake G, Csipke E, Wykes T (2013). Assessing your mood online: acceptability and use of Moodscope. Psychol Med.

[50] Carroll JK, Moorhead A, Bond R, LeBlanc WG, Petrella RJ, Fiscella K (2017). Who uses mobile phone health apps and does use matter? A secondary data analytics approach. J Med Internet Res.

[51] Anguera JA, Jordan JT, Castaneda D, Gazzaley A, Areán Patricia A (2016). Conducting a fully mobile and randomised clinical trial for depression: access, engagement and expense. BMJ Innov.

[52] Corbin J, Strauss A (2014). Basics of qualitative research: Techniques and procedures for developing grounded theory, 4th Edition.

[53] Simonsen J, Friberg K (2014). Collective analysis of qualitative data. Situated design methods.

[54] Fusch P, Ness L (2015). Are we there yet? Data saturation in qualitative research. The Qualitative Report.

[55] Davies W (2016). How are we now? Real-time mood-monitoring as valuation. Journal of Cultural Economy.

[56] McConville C, Cooper C (1998). Personality correlates of variable moods. Personality and Individual Differences.

[57] Beck JS (2021). Cognitive behavior therapy: Basics and beyond.

[58] Shin G, Feng Y, Jarrahi M, Gafinowitz N (2019). Beyond novelty effect: a mixed-methods exploration into the motivation for long-term activity tracker use. JAMIA Open.

[59] Costa Figueiredo M, Caldeira C, Eikey EV, Mazmanian M, Chen Y (2018). Engaging with health data: The interplay between self-tracking activities and emotions in fertility struggles. Proceedings of the ACM on Human-Computer Interaction.

[60] Rooksby J, Rost M, Morrison A, Chalmers M (2014). Personal tracking as lived informatics. Proceedings of the SIGCHI Conference on Human Factors in Computing Systems.

[61] Ayobi A, Sonne T, Marshall P, Cox A (2018). Flexible and mindful self-tracking: Design implications from paper bullet journals. Proceedings of the 2018 CHI Conference on Human Factors in Computing Systems.

[62] Mackay W (1991). Triggers and barriers to customizing software. Proceedings of the SIGCHI Conference on Human Factors in Computing Systems.

[63] Choe E, Lee B, Kay M, Pratt W, Kientz J (2015). SleepTight: Low-burden, self-monitoring technology for capturing and reflecting on sleep behaviors. Proceedings of the 2015 ACM International Joint Conference on Pervasive and Ubiquitous Computing.

[64] Zhang X, Pina L, Fogarty J (2016). Examining unlock journaling with diaries and reminders for in situ self-report in health and wellness. Proceedings of the 2016 CHI Conference on Human Factors in Computing Systems.

[65] Bentley F, Tollmar K (2013). The power of mobile notifications to increase wellbeing logging behavior. Proceedings of the SIGCHI Conference on Human Factors in Computing Systems.

[66] Dockray S, Grant N, Stone AA, Kahneman D, Wardle J, Steptoe A (2010). A comparison of affect ratings obtained with ecological momentary assessment and the day reconstruction method. Soc Indic Res.

[67] Wallbaum T, Heuten W, Boll S (2016). Comparison of in-situ mood input methods on mobile devices. Proceedings of the 15th International Conference on Mobile and Ubiquitous Multimedia.

[68] Highfield T, Leaver T (2016). Instagrammatics and digital methods: studying visual social media, from selfies and GIFs to memes and emoji. Communication Research and Practice.

[69] Schueller SM, Muñoz Rf, Mohr DC (2013). Realizing the Potential of Behavioral Intervention Technologies. Curr Dir Psychol Sci.

[70] Sarkar U, Gourley GI, Lyles CR, Tieu L, Clarity C, Newmark L, Singh K, Bates DW (2016). Usability of Commercially Available Mobile Applications for Diverse Patients. J Gen Intern Med.

[71] Eschler J, Burgess E, Reddy M, Mohr D (2020). Emergent self-regulation practices in technology and social media use of individuals living with depression. Proceedings of the 2020 CHI Conference on Human Factors in Computing Systems.

