# Supplementary material for: Understanding People’s Use of and Perspectives on Mood-Tracking Apps: Interview Study
Source: JMIR Ment Health. 2021 Aug 11;8(8):e29368. doi: 10.2196/29368 (PMC8387890; doi:10.2196/29368)
Supplement: Multimedia Appendix 1 [file mental_v8i8e29368_app1.pdf]

Interview Guide

Welcome: Introductions & Demographics

I'm *[name]* and this is *[second interviewer name]*. I'm a Research Assistant with the [*removed for review*]. Thank you for joining me today, I really appreciate you sharing your time to come in and talk to me. First off, please read through this study information sheet, which explains the purpose of the study and how the data is collected and stored. After you've read through it, I can answer any questions you have.

[Have participant read study information sheet;]

Do you have any questions?

As noted in the study information sheet, I'll be audio recording the interview. Do you agree to participate in this study, and be audio recorded?

Demographic questions

Before we start talking about your experience using apps, I'd like to have you complete this questionnaire; these questions are optional. When you have completed the questions you can turn over the sheet and hand it back to me.

Phone & technology use

Great. Let's get started. Just to remind you, there are no right or wrong answers to any of the questions today. We're really interested in learning about everyone's unique experiences. You are the expert. We are here to understand your perspectives and experiences, so we will not be sharing our opinions. I'll be mostly asking questions and *[second interviewer]* will take some notes. At the end, I'll pass it over to *[second interviewer]* to see if they have any additional questions.

1. First, I'd like to start by learning a little more about how you use technology and your phone in general. How often do you use your smartphone? *[Use probing questions as needed*

As you read in the study information sheet. the purpose of the study is to learn about how and why you have used mood tracking apps, what you found useful & problematic about these apps, and how mood tracking apps can better serve users. I see that you've used [app name - refer to Demographics Sheet].

2. What prompted you to first start using this app?
   PROMPT: Maybe it was a life event, or a suggestion or encouragement from family member or friend.
   If they have used more than one app → in general...

3. Why did you choose the particular app(s)?
   a. Where did you find them/how did you hear about them?

   b. Did you consider other apps when you were choosing an app?

   c. What other app(s) did you consider? Why did you choose this one?
      If they have used more than one app → in general…

4. When do you use the app?

   PROBE: What triggers, cues, or life events make you more likely to open the app?
   PROBE: For example this may be a habit, a need for advice/support, or something which happens in your life which you want to record
   If they have used more than one app → in general…

5. How often do/did you use these apps?

   If they have used more than one app → in general...

6. What do you do with this app? / How do you use this app?
   a. Why those items?
   b. Are there other things you would want to enter instead/in addition?

   PROBE: What types of things do you typically enter about your mood, emotions, thoughts, or feelings?

   PROBE: What do/did you normally enter into the app?

   If they have used more than one app → thinking about the app you liked the most…

7. How do you review the information you enter about your mood?
   a. How do you feel about how the app presents this information back to you?
   b. What questions do you have about your data which the app doesn't answer?

   If they have used more than one app → thinking about the app you liked the most...

8. What did you find helpful about the app?

9. What did you find problematic about the app?

10. If a friend who was interested in tracking their mood came and asked you about this app, what would you tell them?

11. What do you think is missing from this app?

    If they have used more than one app → in general...

If participant used apps in the past but not currently using:

1. Why did you stop using them?
2. Would you want to use it again? Why or why not?

Is there anything else you'd like to tell me about the app which I haven't yet asked about?

Card sorting activity

Protocol

Remember: At each stage, we are interested in **why** the participant categorized something the way they did. **This includes those categorized as not interested.** Be sure to probe and encourage them to explain their choice of category.

1. **Data Entry Screens:** Ask participant to categorize data entry screens into "Interested" and "Not Interested"
   a. Take the "Interested" data entry screens, and have participant categorize these into "Too Specific", "Too General", "Just Right"
2. **Data Review Screens:** Ask participant to categorize data review screens into "Interested" and "Not Interested"
   a. Take the "Interested" data review screens, and have participant categorize these into "Too Complicated", "Too Simple", "Just Right"

Script

Now that we have a sense of the apps that you have used and how you feel about them, I'd like get your thoughts on these cards with screenshots from some mood tracking apps to better understand what would and would not work well for you. To understand this, I'll ask you to categorize some screenshots from mood-tracking apps.

Let's start with these screenshots which show different ways you can enter information into an app. I'll hand you these cards one by one, and you will put it into the "Interested" or "Not interested" category, depending on how you feel about the way the app collects information. Please talk aloud as you're deciding which category each card should go in. The categories are just suggestions. You're welcome to create your own categories or place a screenshot between multiple categories. You're also free to recategorize something at any time.

Let’s start with screen number XX ***[remember to name each screen by number or letter as you hand it over to the participant, for the purposes of the audio recording]***

For each screenshot, make sure a participant answers these questions. They may address them in their think-aloud, rather than a direct question:

- **Why did you place this screenshot in this category?**
- **Why did you feel this category was a better fit than the other categories?**
- **What about this screen would you find useful?**

Remember to probe to understand why participant categorized each card the way they did Once complete, probe to understand why they are not interested in the apps they put in the “Not Interested” category.

So let’s take these apps you put in the “Interested” category and talk a little more about them. Please put these into the categories “Too Specific”, “Too General”, “Just Right”, depending on how you feel about these apps. Again, feel free to talk aloud, I’d really like to understand your reactions and thoughts on these screenshots.

- **Why did you place this screenshot in this category?**
- **Why did you feel this category was a better fit than the other categories?**
- **What about this screen would you find useful?**

Great! Now let’s look at a new set of screenshots. These cards show ways different apps present information back to you, and how you can review the information you have entered. Let’s start the same way as before: please tell me if you are “Interested” or “Not Interested” in how these apps show information, using these categories.

- **Why did you place this screenshot in this category?**
- **Why did you feel this category was a better fit than the other categories?**

- **What about this screen would you find useful?**

So let’s take these apps you put in the “Interested” category and talk a little more about them. We’ll use the categories “Too Complicated”, “Too Simple”, “Just Right” to talk a bit more into these apps. Thinking some more about these apps, please categorize these. Again, feel free to talk aloud, I’d really like to understand your reactions and thoughts on these screenshots.

- **Why did you place this screenshot in this category?**
- **Why did you feel this category was a better fit than the other categories?**
- **What about this screen would you find useful?**
