# Supplementary material for: Understanding People’s Use of and Perspectives on Mood-Tracking Apps: Interview Study
Source: JMIR Ment Health. 2021 Aug 11;8(8):e29368. doi: 10.2196/29368 (PMC8387890; doi:10.2196/29368)
Supplement: Multimedia Appendix 2 [file mental_v8i8e29368_app2.pdf]

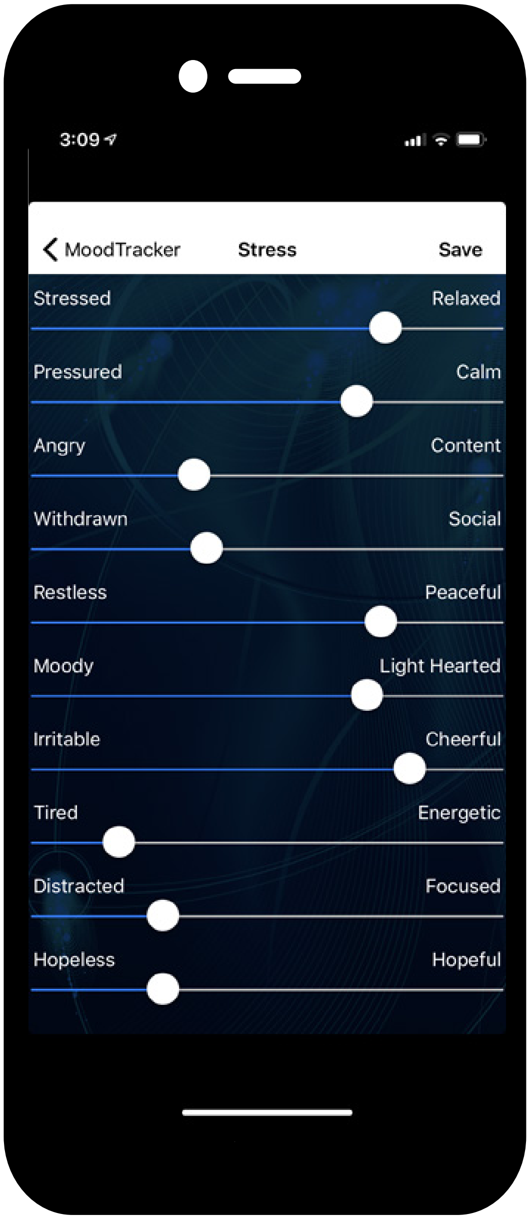

Sliders with text endpoints for many scales, from T2 Mood Tracker

1

Free text entry,
from MoodTrack Diary: Mood Tracker

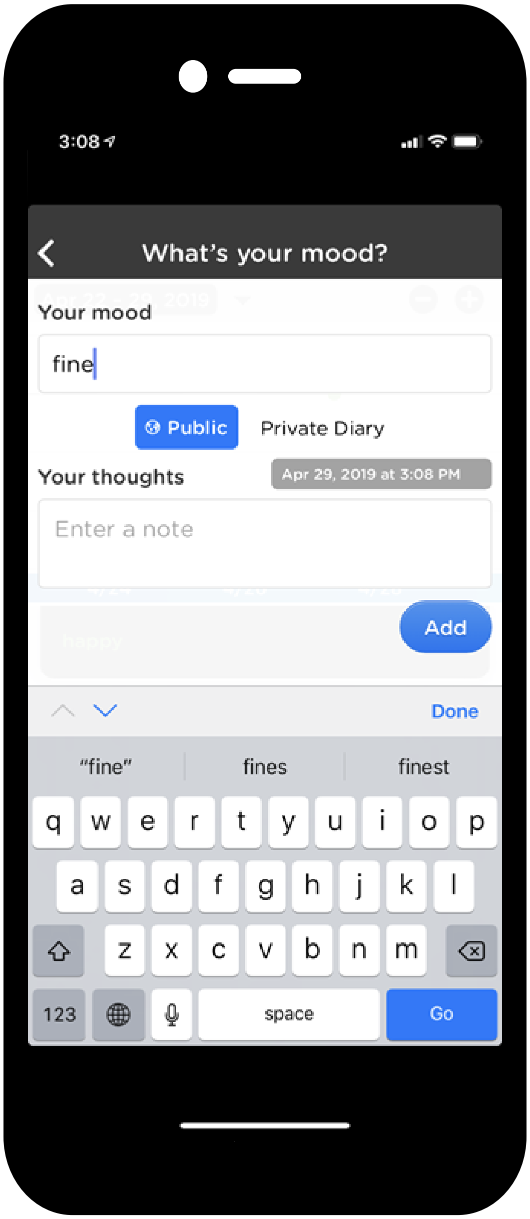

2

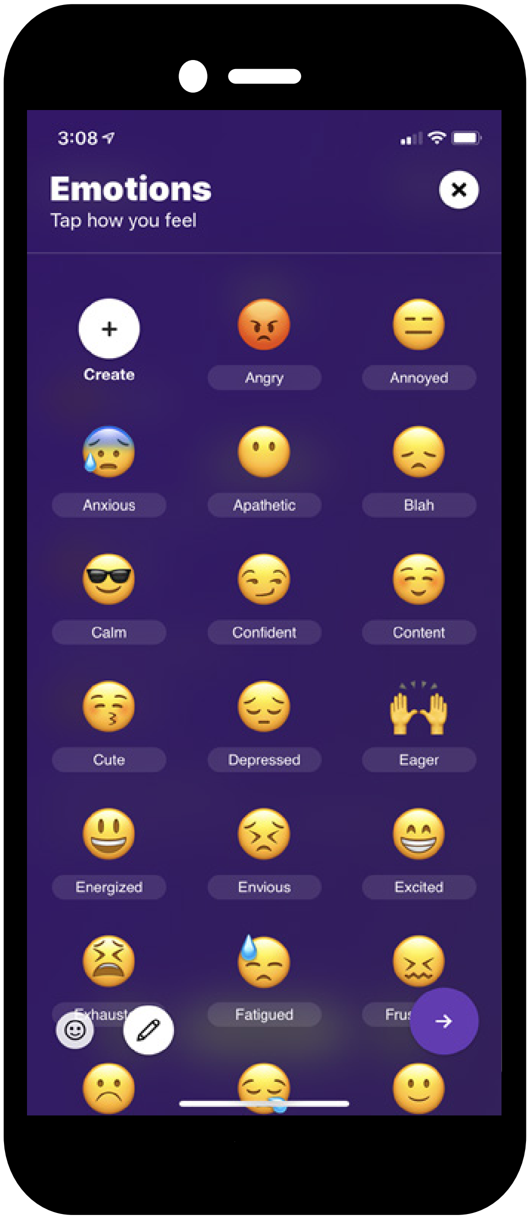

Emoji selection, from
Mood - Journal & Anxiety Chat

3

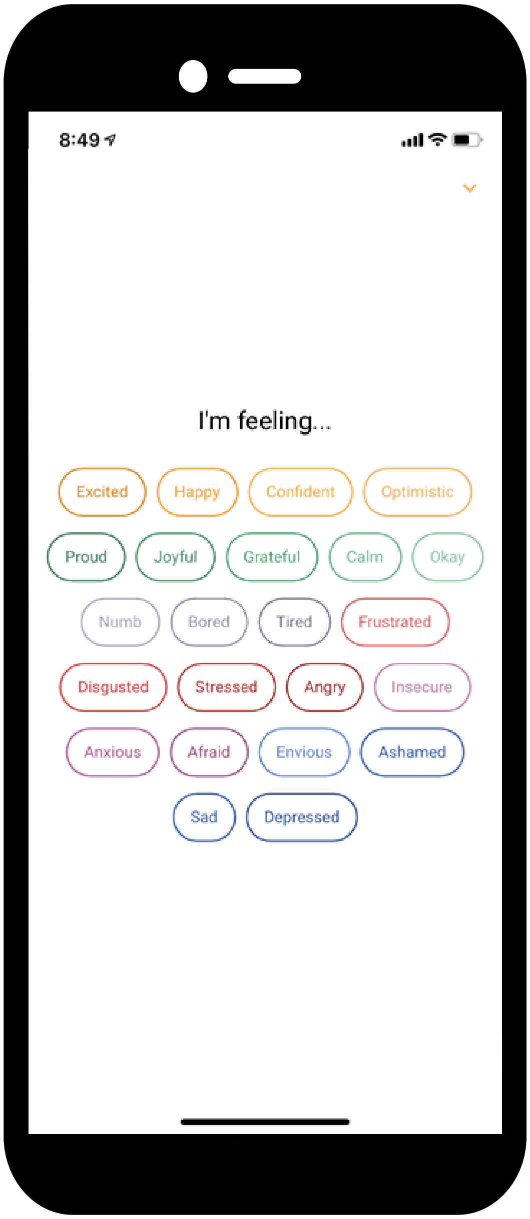

Word selection,
from Youper

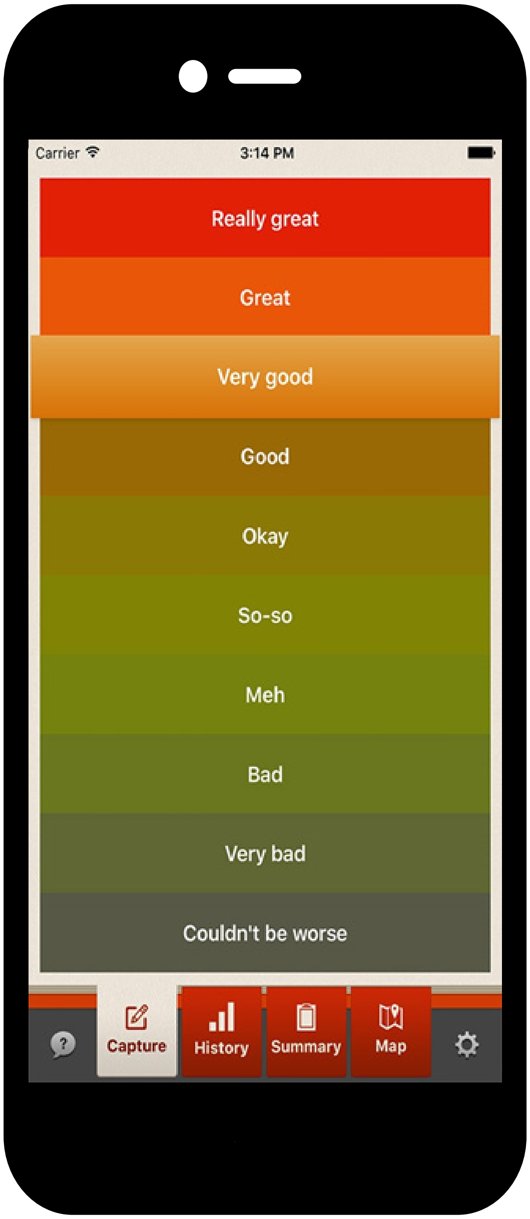

Scale of text description and color, from iMoodJournal

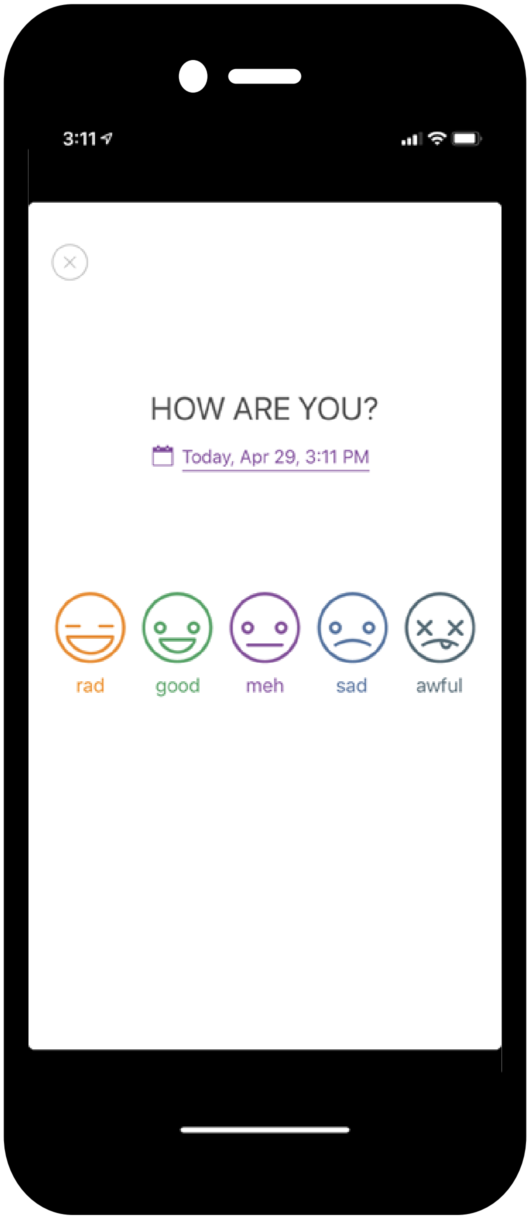

Scale of emoji, from Daylio

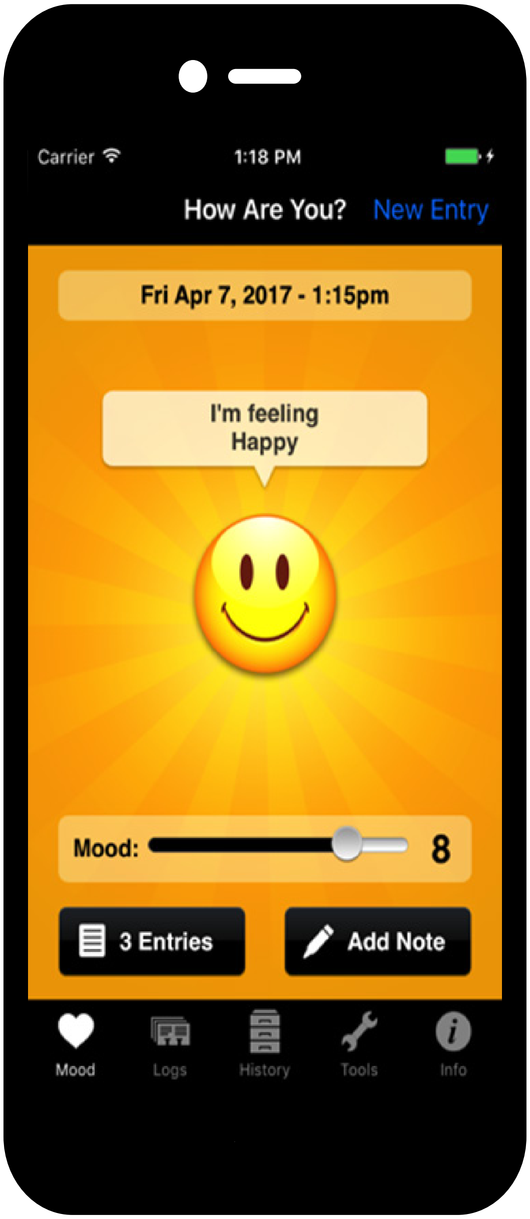

Numbered slider with reflective emoji, from MyMoodTracker
