# Supplementary material for: Understanding People’s Use of and Perspectives on Mood-Tracking Apps: Interview Study
Source: JMIR Ment Health. 2021 Aug 11;8(8):e29368. doi: 10.2196/29368 (PMC8387890; doi:10.2196/29368)
Supplement: Multimedia Appendix 3 [file mental_v8i8e29368_app3.pdf]

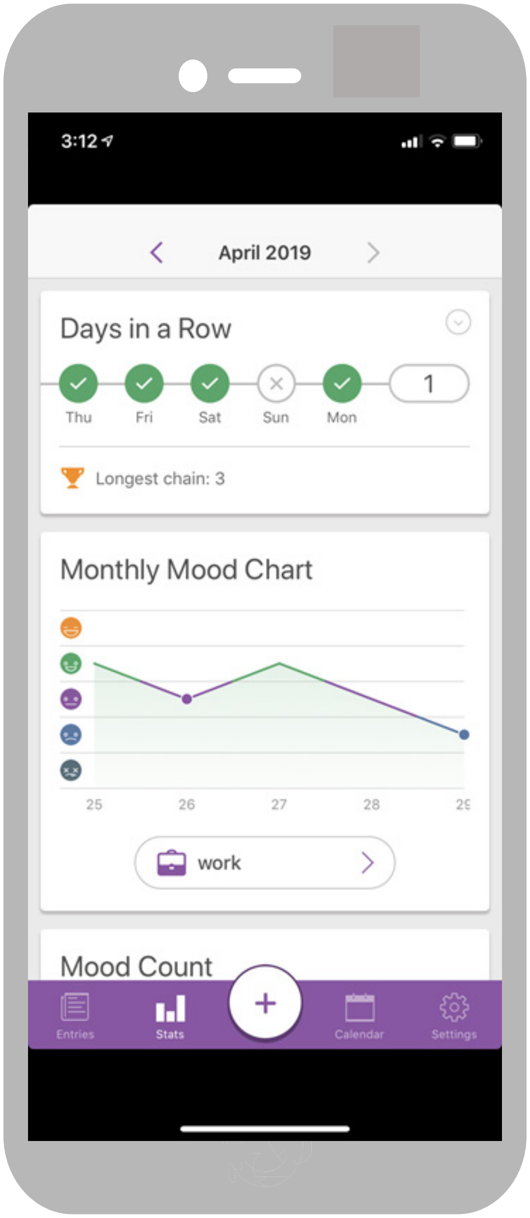

A

Line graph and streaks, from Daylio

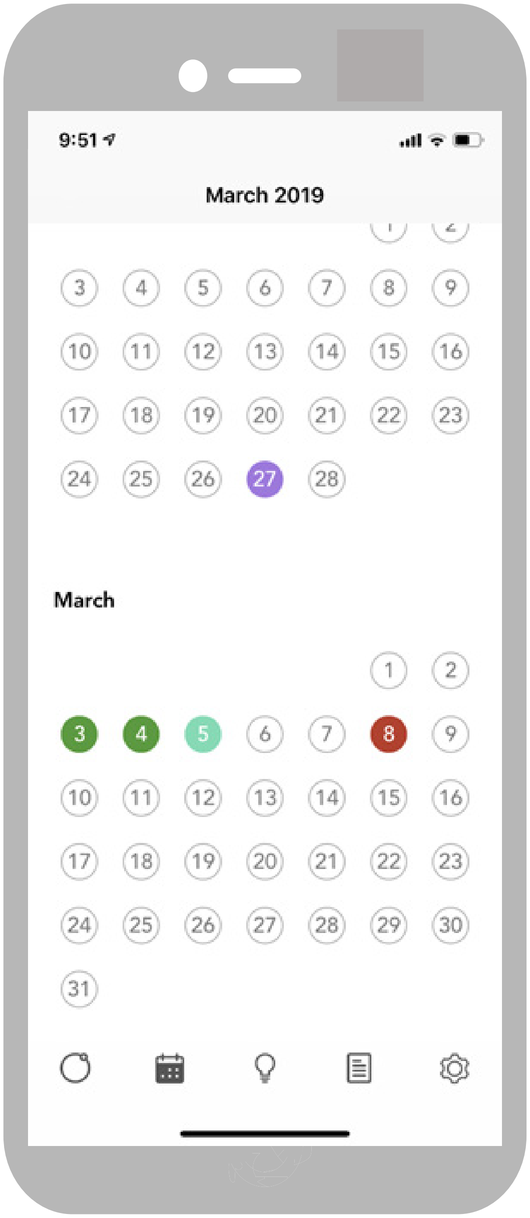

Calendar,
from Vibrant

B

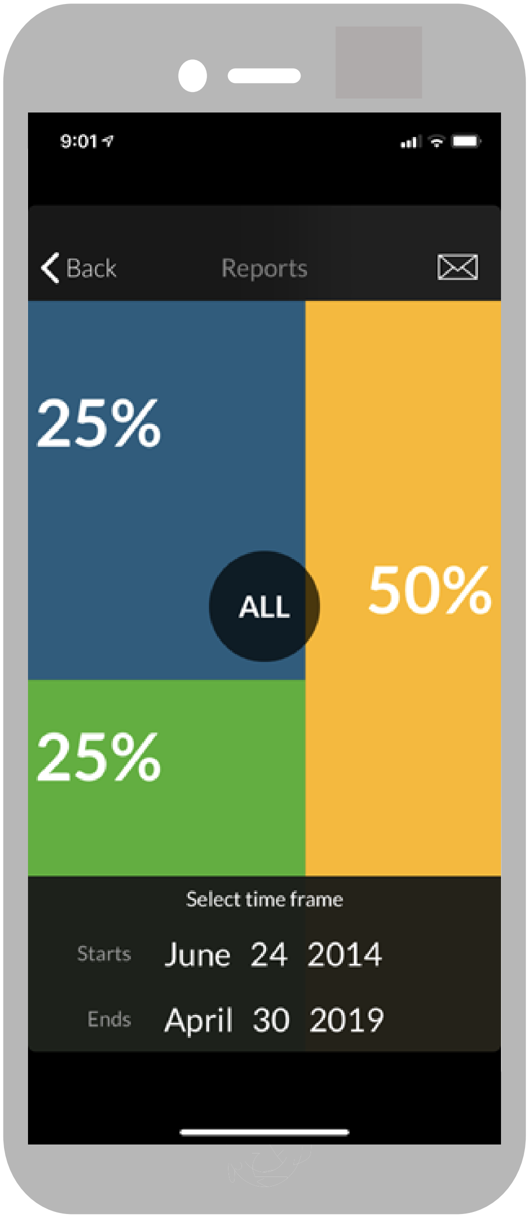

C

Percentage summary of time range, from Mood Meter

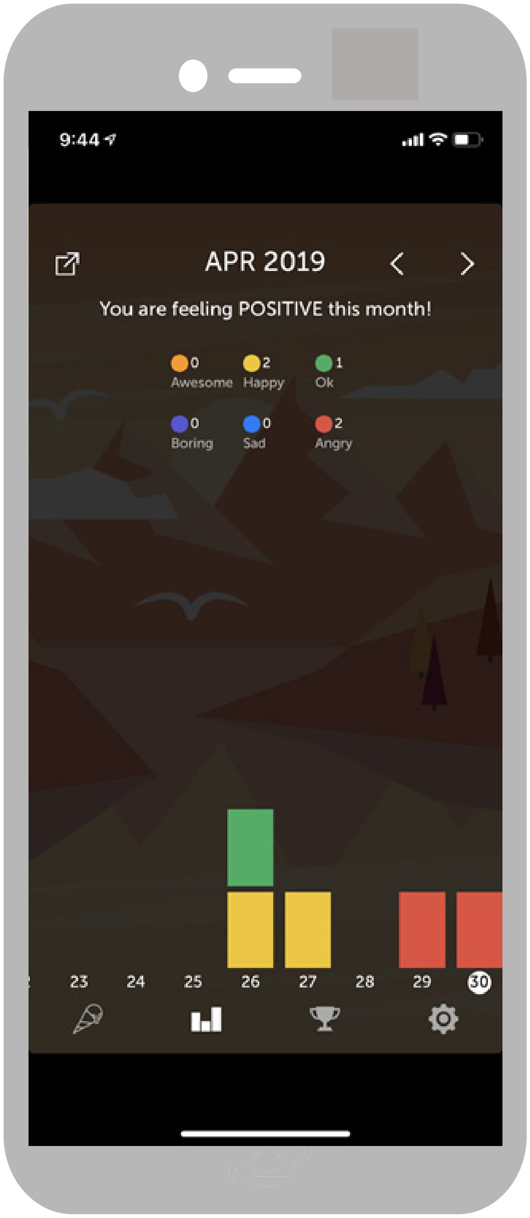

Bar chart,
from Mood D - Your emotional diary

D

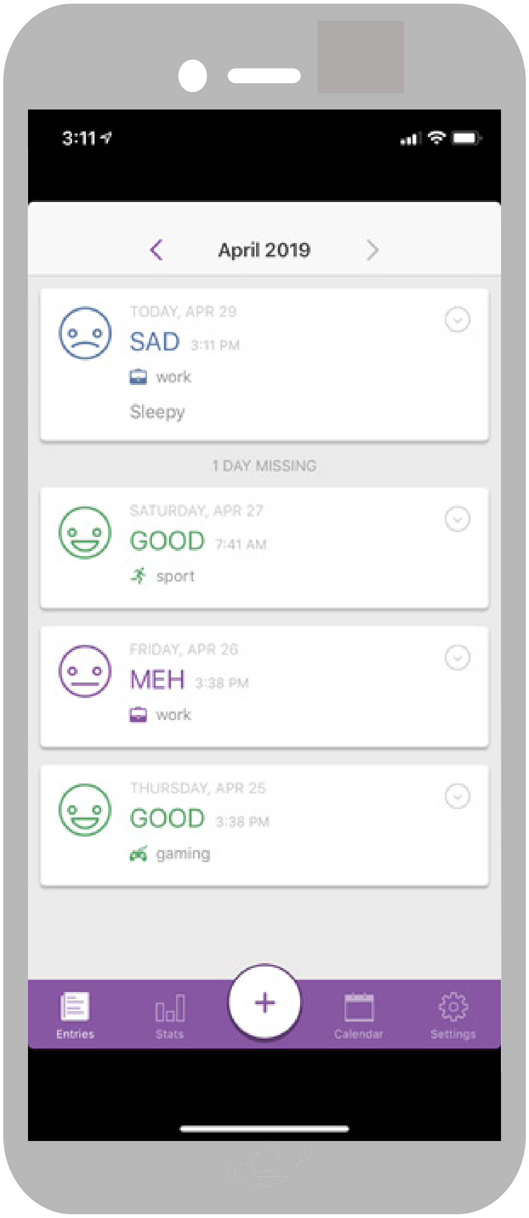

Feed,
from Daylio

E
